# Impact of an internet-based insomnia intervention on suicidal ideation and associated correlates in veterans at elevated suicide risk

**DOI:** 10.1093/tbm/ibae032

**Published:** 2024-06-12

**Authors:** Sarra Nazem, Shengnan Sun, Sean M Barnes, Lindsey L Monteith, Trisha A Hostetter, Jeri E Forster, Lisa A Brenner, Hanga Galfalvy, Fatemeh Haghighi

**Affiliations:** Dissemination and Training Division, National Center for PTSD, Palo Alto, CA, USA; Department of Neuroscience, Icahn School of Medicine, New York, NY, USA; General Medical Research (GMR), James J. Peters VA Medical Center, Bronx, NY, USA; VA Rocky Mountain Mental Illness Research, Education and Clinical Center (MIRECC), Aurora, CO, USA; Department of Psychiatry, University of Colorado School of Medicine, Aurora, CO, USA; VA Rocky Mountain Mental Illness Research, Education and Clinical Center (MIRECC), Aurora, CO, USA; Department of Psychiatry, University of Colorado School of Medicine, Aurora, CO, USA; Department of Physical Medicine and Rehabilitation, University of Colorado School of Medicine, Aurora, Co, USA; VA Rocky Mountain Mental Illness Research, Education and Clinical Center (MIRECC), Aurora, CO, USA; VA Rocky Mountain Mental Illness Research, Education and Clinical Center (MIRECC), Aurora, CO, USA; Department of Physical Medicine and Rehabilitation, University of Colorado School of Medicine, Aurora, Co, USA; VA Rocky Mountain Mental Illness Research, Education and Clinical Center (MIRECC), Aurora, CO, USA; Department of Psychiatry, University of Colorado School of Medicine, Aurora, CO, USA; Department of Physical Medicine and Rehabilitation, University of Colorado School of Medicine, Aurora, Co, USA; Department of Neurology, University of Colorado School of Medicine, Aurora, CO, USA; Department of Psychiatry, Columbia University, New York, NY, USA; Department of Biostatistics, Columbia University, New York, NY, USA; Department of Neuroscience, Icahn School of Medicine, New York, NY, USA; General Medical Research (GMR), James J. Peters VA Medical Center, Bronx, NY, USA

**Keywords:** internet intervention, insomnia, sleep treatment, suicide, technology, veteran

## Abstract

Improving public health approaches to suicide prevention requires scalable evidence-based interventions that can be easily disseminated. Given empirical data supporting the association between insomnia and suicide risk, internet-delivered insomnia interventions are promising candidates to meet this need. The purpose of this study was to examine whether an unguided internet-delivered cognitive-behavioral therapy for insomnia (iCBT-I) improved insomnia severity, suicidal ideation (SI), and suicide risk correlates (depression, post-traumatic stress disorder, anxiety, hostility, belongingness, hopelessness, agitation, irritability, concentration) in a sample of veterans. Secondary data analysis of Operation Enduring Freedom, Operation Iraqi Freedom, and Operation New Dawn veterans (*n* = 50) with clinically significant insomnia and elevated SI drawn from a larger randomized controlled trial (RCT) of an iCBT-I, Sleep Healthy Using the Internet (SHUTi). Two-sample *t*-tests or Wilcoxon rank sum tests were used to evaluate between-group differences (SHUTi vs. Insomnia Education Website control) in symptom improvement from baseline to post-intervention. SHUTi participants experienced a significant improvement in insomnia severity (*P* < .001; *d* = −1.08) and a non-significant with small (subthreshold medium) effect size reduction of SI (*P* = .17, *d* = 0.40), compared to control participants. Significant improvement in hopelessness was observed (medium effect size), with non-significant small to medium effect size reductions in most remaining suicide risk correlates. Self-administered iCBT-I was associated with improvements in insomnia severity in veterans at elevated risk for suicide. These preliminary findings suggest that SI and suicide risk correlates may improve following an iCBT-I intervention, demonstrating the need for future well-powered iCBT-I RCTs targeted for populations at elevated suicide risk.

## Teaser

Veterans with suicidal ideation who received internet-delivered cognitive-behavioral therapy for insomnia experienced improvements in insomnia severity and hopelessness.

**Implications**

**Practice:** Internet-delivered cognitive-behavioral therapy for insomnia can be utilized to improve insomnia severity and hopelessness in veterans with suicidal ideation.

**Policy:** Leveraging technology to provide evidence-based insomnia intervention to those at risk for suicide is a promising public health approach to suicide prevention.

**Research:** Future research designed to examine suicide risk correlates in large-scale internet- and digital-based cognitive-behavioral therapy for insomnia interventions is necessary to inform scalable interventions for at-risk populations.

## INTRODUCTION

Insomnia is a significant public health and clinical problem. From 1999 to 2010, outpatient visits due to insomnia symptoms increased by 13% [1] and diagnoses of insomnia in adults increased from 11.9% to 15.5% between 2000 and 2010 [2]. Insomnia and related sleep disturbances have been recognized for decades by the Veterans Health Administration (VHA) as highly prevalent and persistent problems [3]. From 2000 to 2010, there was a 7-fold increase in diagnoses of insomnia among veterans of all ages receiving care in VHA [4, 5]. Between FY2012 and FY2018, the prevalence of insomnia diagnoses in VHA continued to steadily increase from 7.4% to 11.8% [6]. Further, in a nationally representative sample of US veterans, approximately one-third endorsed symptoms consistent with clinical or subthreshold insomnia [7].

Elevated rates of insomnia disorder and symptoms are especially concerning given that insomnia is associated with increased risk for suicidal ideation (SI) and self-directed violence (SDV), across community, college, and clinical samples (e.g. inpatient samples, samples comprised of individuals with psychiatric symptoms or elevated suicide risk) and a broad range of methodological designs (i.e. cross-sectional, prospective, longitudinal [8–15]). In a recent meta-analysis, insomnia was associated with an increased relative risk (RR) for SI, suicide attempt, and suicide mortality [RR = 2.84, 95% Confidence Interval (CI): 2.44–3.31], and this association remained significant after adjusting for potential confounders, such as depression and anxiety (RR = 1.98, 95% CI: 1.63–2.41 [16]). Meta-analytic work also suggests that individuals with comorbid psychiatric diagnoses [e.g. major depressive disorder, posttraumatic stress disorder (PTSD), panic disorder] and insomnia symptoms are more likely to endorse SI and engage in SDV (odds ratio = 2.66, 95% CI: 1.74–4.07), compared to individuals with psychiatric diagnoses and no insomnia-related symptoms [12]. Further, SI and SDV are linked to complaints and impairments commonly seen among those with insomnia, such as self-reported short sleep duration [17, 18], low sleep efficiency (the proportion of time in bed spent asleep [19]), poor sleep quality [20–22], sleep variability [23], and nocturnal wakefulness [15, 24]. Notably, *persistent* symptoms of insomnia are associated with increased odds of depression and SI [25], suggesting the need for early identification and treatment to reduce future negative outcomes, such as SDV.

Due to its association with suicide risk, insomnia is a promising suicide prevention target, particularly given that it is a *modifiable* risk factor [26]. Although there are many challenges that underlie suicide prevention (e.g. stigma), one major obstacle is that many risk factors for suicide—childhood and/or combat trauma histories, demographic characteristics, suicide attempt history, and genetic risk factors—are not readily modifiable. In contrast, because insomnia is dynamic, time-varying, and typically proximal to the timing of suicidal states and behaviors [10], it is a promising modifiable upstream and downstream target for suicide prevention.

A substantial body of research supports the effectiveness of cognitive-behavioral therapy for insomnia (CBT-I) for the treatment of insomnia, resulting in the recognition of CBT-I as the first-line method of treatment (National Institutes of Health consensus) [27]. Compared to sleep hygiene, which aims to reduce sleep problems by providing psychoeducation on reducing stimulating activities prior to sleep and establishing a sleep regimen, among other practices, CBT-I targets maladaptive behaviors and dysfunctional thoughts believed to perpetuate sleep problems using five primary treatment components (sleep restriction, stimulus control, cognitive restructuring, sleep hygiene, relapse prevention [28]). CBT-I is associated with high rates of treatment response and remission, with moderate to very large effect sizes [29–32] and low dropout rates [33]. Improvements in insomnia are typically achieved within 4–6 weeks and are maintained over time [34–36].

Due to mounting evidence on the effectiveness of CBT-I, VHA created a national dissemination plan for CBT-I as part of its larger initiative to make evidence-based psychotherapies more widely available to veterans. In the VHA national rollout of CBT-I, at post-treatment, 73% of veterans achieved Insomnia Severity Index scores (ISI [37, 38]) below the cut-off for moderately severe insomnia symptoms (ISI < 15), and 35% no longer met the criteria for insomnia (ISI < 8), demonstrating gains similar to those observed in non-veteran populations. CBT-I has also been found to be effective for individuals with insomnia and comorbid disorders, reducing both insomnia severity and the severity of comorbid disorders, including PTSD, anxiety/depression, alcohol and substance use disorders, and SI in both civilian and military samples [26, 29, 31, 32, 37, 39–44]. In a recent proof-of-concept randomized controlled trial (RCT) designed specifically for VHA primary care patients with SI, insomnia, and either major depressive disorder or PTSD, Pigeon and colleagues [45] found that brief CBT-I was associated with reductions in insomnia, depression, and SI (Hedge’s *g* = 1.91, 1.16, 0.26, respectively), compared to those randomized to treatment as usual, confirming that CBT-I positive outcomes extend to populations at elevated risk for suicide.

In spite of this, only a small percentage of individuals with insomnia disorder receive treatment for their symptoms [46] due to a lack of trained clinicians and overreliance on in-person models of delivering care. In-person treatment formats create barriers to receiving and/or adhering to treatment visit schedules due to travel/distance to the clinic, conflicts with work and family schedules, and childcare availability. Over the past several years, internet- and mobile/digital-based CBT-I interventions have been developed to reduce these barriers, offering a potentially efficacious way for individuals to receive evidence-based treatment that they might not receive in a timely manner. Modeled on efficacious CBT-I treatment, internet- and mobile/digital-based CBT-I [47–51] has been found to improve insomnia symptom severity, sleep parameters, and subjective sleep quality, with comparable effect sizes to those found with face-to-face CBT-I (i.e. Hedges’s *g* ranging from 0.21 to 1.09 [52]). One of the most empirically examined internet-delivered CBT-I (iCBT-I) options is Sleep Healthy Using the Internet (SHUTi [53–58]). Data from several rigorous clinical trials of SHUTi, examined across a variety of patient groups, consistently show that SHUTi is associated with significant and clinically meaningful improvements in sleep outcomes and co-occurring symptoms [53, 54, 56–63].

To mitigate suicide risk, public health approaches to suicide prevention must incorporate multilevel strategies. Leveraging technology to provide evidence-based insomnia intervention to those at risk holds incredible promise as both a selective intervention (i.e. targeting an at-risk group, those with insomnia disorder, who are not yet reporting SI) and as an indicated intervention (i.e. targeting the highest risk group, those with insomnia disorder who are also displaying signs of suicide risk [64]).

Furthermore, using technology to deliver evidence-based insomnia intervention expands dissemination potential, a critical need in current suicide prevention [65] approaches. Despite these needs, only one identified study has examined a digital CBT-I in a population at elevated risk for suicide. Utilizing data from a larger RCT, Kalmbach and colleagues [66] found that patients with baseline SI randomized to receive digital CBT-I reported lower SI rates at post-treatment and at the 1-year follow-up relative to controls. To further inform the development and implementation of indicated interventions, and to build upon Kalmbach and colleagues’ work, we examined whether an alternate iCBT-I, SHUTi, improved insomnia severity, SI, and suicide risk correlates (depression, post-traumatic stress disorder, anxiety, hostility, belongingness, hopelessness, agitation, irritability, concentration) in a sample of veterans with clinically significant insomnia and elevated SI drawn from a larger RCT of SHUTi. In this secondary data analysis, we hypothesized that participants randomized to SHUTi would report a greater reduction in insomnia severity and SI compared to participants who were randomized to an Insomnia Education Website control (IEW). Secondarily, we hypothesized that SHUTi participants would report a greater reduction in all suicide risk correlates compared to IEW participants.

## Materials and Methods

### Participants and procedures

Our sample was comprised of participants drawn from a single-blind, two-group (SHUTi vs. IEW) RCT designed to evaluate the efficacy of SHUTi in improving insomnia symptoms in Operation Enduring Freedom (OEF), Operation Iraqi Freedom (OIF), and Operation New Dawn (OND) veterans [67]. Recruitment for the RCT included targeted mailings sent to veterans with recently diagnosed sleep problems, as identified by diagnostic codes within the VA Corporate Data Warehouse (CDW).

Eligibility for the RCT included the following: current DSM 5 insomnia diagnosis (American Psychiatric Association, 2013), US military veteran, receiving care in the Mountain West VA region, reliable access to the Internet, proficient in English, and able to provide informed consent. Exclusion criteria included: current participation in other interventional research studies, untreated sleep disorder besides insomnia, currently receiving psychological treatment for insomnia, medication change designed to improve, or impact sleep in the past 3 months, history of bipolar I disorder, schizophrenia, schizoaffective disorder, psychotic disorder, and other criteria contraindicated with sleep restriction (e.g. untreated seizures, pregnancy, irregular shift work, significant cognitive impairment, current non-alcohol substance use disorder).

After being screened by phone for eligibility, 289 veterans were eligible, 250 of whom provided informed consent and enrolled in the RCT (23 were lost-to-contact, 15 decided not to enroll, and 1 missed the enrollment window). After enrollment, participants were informed that they had to complete 10/14 sleep diaries to move forward in the study; 15 (6.0%) of enrolled participants did not complete the sleep diary requirement. Baseline assessments were unlocked online once the sleep diary requirement was fulfilled, with randomization following the completion of the baseline assessment. Three participants did not complete the baseline assessment, resulting in 231 (92.4%) participants being randomized. Randomization was stratified by suicide attempt history (yes/no) to ensure equal representation between groups on this suicide risk variable.

Participants had access to their assigned intervention materials for 9 weeks, after which participants were invited to complete their post-intervention assessment. A total of 189 participants (81.8%) completed the post-intervention assessment (*n* = 91 SHUTi, *n* = 98 IEW). To determine whether SHUTi is associated with improvements in insomnia symptom severity, SI, and other suicide risk correlates in an at-risk sample for suicide, the analytic sample was restricted to participants with baseline clinically significant insomnia (defined by an ISI total score ≥15) and baseline moderate to elevated SI [defined by an Adult Suicidal Ideation Questionnaire (ASIQ) total score ≥14], who also completed post-intervention assessments. A total of 50 participants met these criteria (*n* = 23 SHUTi, *n* = 27 IEW).

All data were collected online, and all participants provided informed consent prior to study enrollment. The study design and protocol were approved by the Colorado Multiple Institutional Review Board at the University of Colorado Anschutz Medical Campus . Regulatory approval was also obtained from supporting agencies (Rocky Mountain Regional Veterans Affairs Medical Center Research and Development Committee and US Army Medical Research and Development Command Human Research Protection Office).The study was conducted according to the guidelines of the Declaration of Helsinki.

### Interventions

#### SHUTi

SHUTi is a self-guided, interactive, and tailored internet-based program modeled on the primary tenants of face-to-face CBT-I (sleep restriction, stimulus control, cognitive restructuring, sleep hygiene, relapse prevention). Intervention content is delivered through six “cores.” Users obtain access to a new core on a time and event-based schedule (e.g. 7 days after completion of the previous core). The SHUTi program relies on user-entered online sleep diaries to track progress and tailor treatment recommendations (i.e. assign a “sleep restriction” window). Each core acts as an online analog for the weekly sessions typically used when delivering CBT-I in a face-to-face format, following the same general structure: (i) core objectives (what will be learned and why this information is important); (ii) review of previous week’s homework and sleep diary data; (iii) new intervention material; (iv) assignment of homework (treatment strategies for the coming week); and (v) a summary of the core’s main points. Intervention content is enhanced through a variety of interactive features, including personalized goal setting, graphical feedback based on inputted symptoms, animations/illustrations to enhance comprehension, quizzes to test user knowledge, patient vignettes, and video-based expert explanations. Automated emails are sent to encourage program adherence.

#### IEW

Informed by what typically constitutes “treatment as usual” for patient education websites targeting insomnia (i.e. sleep hygiene), the IEW includes the following: (i) content presented all at once; (ii) treatment information about insomnia symptoms, diagnosis, and differential diagnosis of insomnia, etiology, the natural history of insomnia and its prognosis, and cognitive-behavioral treatment strategies; (iii) printable documents (including sleep diaries and insomnia severity assessments); (iv) FAQ section which provides answers to frequently encountered patient problems; and (v) automated email prompts to encourage users to return to the site. The IEW has been used extensively in SHUTi trials.

### Measures

#### Demographic form

Basic information about participant demographics (e.g. age, gender, race) was collected at baseline.

#### Insomnia disorder

The insomnia disorder module from the Structured Clinical Interview for DSM-5 [68] (SCID) was utilized to verify that each participant met the current diagnostic criteria for insomnia disorder. Research assistants received extensive training on the SCID and how to use a semi-structured interview focused on clinical sleep history to inform differential diagnoses (e.g. untreated sleep apnea, periodic limb movement disorder, parasomnia, circadian rhythm disorder) necessary to determine RCT eligibility; cases were regularly reviewed by the team and first author/principal investigator to ensure accuracy.

#### *Insomnia Severity Index* [69, 70]

The ISI is a 7-item self-report scale that assesses insomnia symptoms over the past 2 weeks. Each item is scored on a 0–4 scale with total scores ranging from 0 to 28; each 7-point increment is associated with increasing levels of insomnia severity. The ISI has been shown to have adequate test–retest reliability over 3 months, and concurrent validity with sleep diaries and polysomnography [69].

#### *Adult Suicidal Ideation Questionnaire* [71]

The ASIQ is a 25-item self-report instrument designed to estimate a respondent’s current level of SI. Each ASIQ item is rated on a 7-point scale indicating the frequency of each item over the past month (e.g. almost every day, about once a month), with scores ranging from 0 to 150. The ASIQ demonstrates high test–retest reliability and internal consistency in clinical and nonclinical samples [72].

#### *Beck Anxiety Inventory* [73, 74]

The Beck Anxiety Inventory (BAI) is a 21-item, self-report rating inventory that measures subjective, somatic, and panic-related symptoms of anxiety during the past week. Each item is rated on a 4-point Likert scale, with scores ranging from 0 to 63; higher numbers suggest greater anxiety severity.

#### *Beck Depression Inventory-II* [75]

The Beck Depression Inventory-II (BDI-II) is a 21-item, self-report rating inventory that measures characteristic attitudes and symptoms of depression over the past 2 weeks. Each item is rated on a 4-point Likert scale, with scores ranging from 0 to 63; higher numbers suggest greater depression severity. Item-level analyses examined agitation (item 11), concentration difficulty (item 19), and irritability (item 17).

#### *Military Suicide Research Consortium Common Data Elements* [76]

The Military Suicide Research Consortium Common Data Elements (MSRC CDEs) is a large battery reflecting a collection of items from instruments assessing suicide-related constructs. Abbreviated sets of items comprising the MSRC CDEs have demonstrated adequate internal consistency and moderate-to-strong correlations with original, full-length measures [77].

In the present study, we administered the MSRC CDE (V2.3; May 2017) to assess hopelessness (MSRC CDE items 21–25; from the Beck Hopelessness Scale [78]; higher scores reflect greater levels of hopelessness; items rated as true or false; total scores range from 0 to 5); hostility (items 62–68; from the Hostility Buss Perry Scale [79]; higher scores indicate greater levels of hostility; statements rated on a 1–5 scale; total scores range from 7 to 35); and belongingness (items 26–30; corresponds to a subset of items from the Interpersonal Needs Questionnaire—Thwarted Belongingness Subscale [80]; higher scores reflect a greater degree of belongingness; statements are rated on a 7-point Likert scale; total scores range from 5 to 35).

A single item (i.e. “How many times in your lifetime have you made an attempt to kill yourself during which you had at least some intent to die?”) was used to ascertain lifetime suicide attempts, included in the MSRC CDE from the Beck Scale for Suicidal Ideation [81] (BSS).

#### *Post-traumatic Stress Disorder Checklist for DSM-5* [82, 83]

The Post-traumatic Stress Disorder Checklist for DSM-5 (PCL-5) is a 20-item self-report measure that assesses the severity of PTSD symptoms in the past month. Each item is scored on a 5-point scale, ranging from 0 (*not at* all) to 4 (*extremely*), with total scores ranging from 0 to 80.

### Data analysis

Statistical analyses were performed in the statistical language R version R 4.2.1 [84]. Baseline demographic characteristics of the sample (*n* = 50) were compared between the SHUTi and IEW groups using a two-sample *t*-test, Chi-squared test, or Fisher’s exact test, as appropriate, with corresponding *P* values reported.

Score difference (i.e. improvement in symptoms) was calculated as post-intervention assessment score minus baseline assessment score for the ISI, ASIQ, BDI-II, PCL-5, BAI, MSRC CDE Hostility, and MSRC CDE Hopelessness. Since higher scores denote symptom improvement on the MSRC CDE Belonging, the difference in baseline assessment minus post-intervention assessment score was used. For item-wise analysis of the three BDI-II items, score differences were tested as binary variables by taking the difference in post-intervention assessment minus baseline assessment score; scores greater than zero were defined as “improved.”

The differences in scores for all measures using a continuous scale were graphed by group and inspected for outliers. Outliers, defined as values outside 1.5 times the interquartile range above the 3rd and below the 1st quartiles, were winsorized—censored to the nearest non-outlier value to avoid deleting several observations from the analysis [85].

In testing the effect of treatment on the outcomes investigated, two-sample comparisons were performed using a *t*-test or Wilcoxon rank sum test, as appropriate, for continuous scales. The effect size of the difference between SHUTi and IEW was reported via Cohen’s *d* (or robust Cohen’s *d* when a non-parametric test was performed). Effect sizes were compared to thresholds as follows: |*d*| (absolute value of *d*) ≥ 0.8 for large, |*d*| = 0.5 for medium, 0.4 ≥ |*d*| < 0.5 for small-medium, |*d*| = 0.2 for small effect sizes. To assess if baseline score affected group differences in symptom improvement, we used linear models, including the baseline score as a covariate, and assessed group differences across continuous outcomes within these models (*t*-test). Chi-square tests were used to compare group differences on the improvement of the three BDI-II items, with odds ratios reported. Within-group differences (SHUTi or IEW) were tested separately using one-sided *t*-tests or Wilcoxon signed-rank tests for all continuous outcomes.

## Results

There were no significant differences between groups on demographic or baseline characteristics including age, gender, race, ethnicity, and clinical assessments (Table 1). Most participants were male (70%) and White (69%). As expected in this high-risk cohort, a large percentage of participants (46% of the overall sample) reported a history of at least one suicide attempt. Because of the small sample size and non-significant between-group differences, demographic characteristics were not adjusted for in any of the statistical analyses reported below.

**Table 1:** Demographic and baseline clinical characteristics

	SHUTi (*n* = 23)	IEW (*n* = 27)	*P* value
	Mean (SD) or *n* (%)	Mean (SD) or *n* (%)	
Age	40.17 (7.84)	37.74 (7.29)	.26
Gender			
Male	15 (65%)	20 (74%)	.71
Female	8 (35%)	7 (26%)	
Race^a^			
White	16 (73%)	17 (65%)	.96
Black/African American	2 (9%)	2 (8%)	
Mixed Race	1 (5%)	3 (12%)	
Native American/Alaskan Native	1 (5%)	2 (8%)	
Other	2 (9%)	2 (8%)	
Hispanic	5 (22%)	8 (30%)	.76
History of suicide attempt	11 (48%)	12 (44%)	>.99
ISI	19.35 (2.82)	18.96 (3.42)	.67
ASIQ^b^	32.78 (19.60)	31.93 (26.15)	.32
BDI-II	29.96 (10.80)	28.96 (14.30)	.79
PCL-5	47.04 (12.72)	45.85 (19.78)	.80
BAI	22.30 (10.50)	24.26 (15.45)	.61
CDE Hopelessness^b^	2.78 (1.98)	2.22 (1.67)	.25
CDE Hostility	21.17 (5.96)	21.89 (5.78)	.67
CDE Belongingness	20.70 (7.19)	20.93 (7.26)	.91
BDI-II 17: Irritability	1.57 (0.79)	1.52 (1.12)	.86
BDI-II 19: Concentration^b^	1.78 (0.80)	1.56 (0.97)	.33
BDI-II 11: Agitation	1.65 (0.83)	1.59 (0.97)	.92

ASIQ, Adult Suicidal Ideation Questionnaire; BAI, Beck Anxiety Inventory; BDI-II, Beck Depression Inventory II; CDE, Military Suicide Research Consortium Common Data Elements; IEW, Insomnia Education Website; ISI, Insomnia Severity Index; PCL-5, Post-traumatic Stress Disorder Checklist for DSM-5; SHUTi, Sleep Healthy Using the Internet.

^a^ *n* = 22 SHUTi, *n* = 26 IEW.

^b^Wilcoxon rank sum test.

### Efficacy of SHUTi on insomnia severity and SI

**Figure 1:**
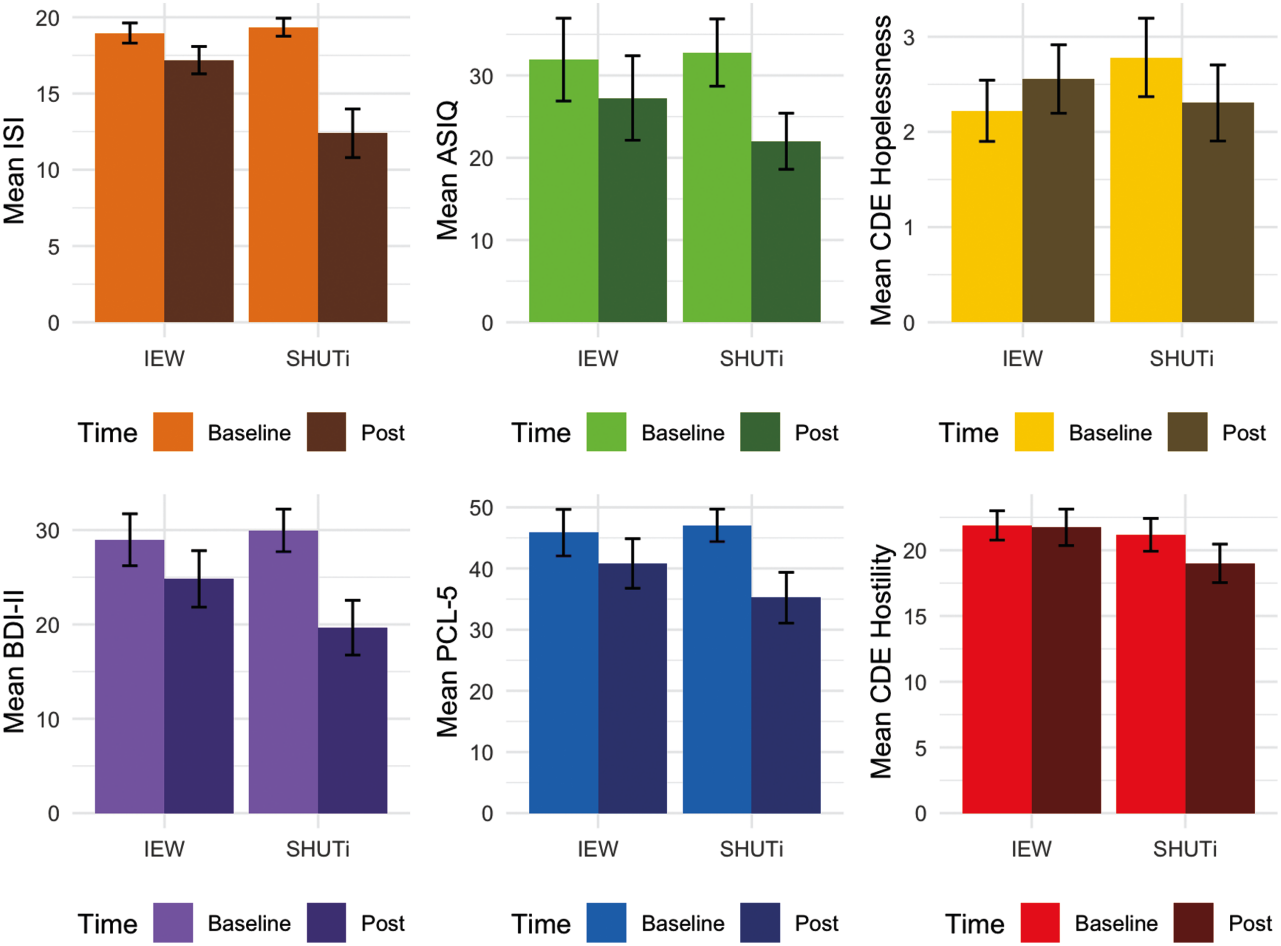
Bar plots showing the average score and standard error (SE) bar for: (A) ISI, (B) ASIQ, (C) CDE Hopelessness, (D) BDI-II, (E) PCL-5, (F) CDE Hostility at baseline and post-intervention assessment for SHUTi and IEW. ASIQ, Adult Suicidal Ideation Questionnaire; BDI-II, Beck Depression Inventory, second edition; CDE, Military Suicide Research Consortium Common Data Elements; IEW, Insomnia Education Website; ISI, Insomnia Severity Index; PCL-5, PTSD Checklist for DSM-5; SHUTi, Sleep Healthy Using the Internet.

Among our high-risk cohort, significantly greater improvement in ISI was observed in the SHUTi group vs. IEW group (*t*_48_ = 3.73, *P* < .001, Fig. 1A and Table 2), with a large effect size (*d* = −1.08). On average, the SHUTi group yielded 5.33 points more reduction in the ISI than the IEW group (Table 2). No statistically significant between-group difference was found in the ASIQ (*t*_48_ = 1.39, *P* = .17, Fig. 1B and Table 2), although the effect size estimate was small-medium (*d* = −0.40). In absolute terms, the SHUTi group achieved a greater reduction in ASIQ scores vs. IEW group by 3.19 points (Table 2). The significance for ISI and ASIQ results remained consistent when accounting for the effect of their corresponding baseline score using a linear model. Within-group analysis for both groups are shown in Supplementary Table S1, with significant large effect sizes observed for both ISI and ASIQ in the SHUTi group and significant but small-medium effect sizes (ISI, ASIQ) observed for the IEW group.

**Table 2:** Change in clinical outcomes at post-intervention between groups

	SHUTi (*n* = 23)	IEW (*n* = 27)	SHUTi vs. IEW		
	Mean (SD)	Mean (SD)	Test statistics	*P*	*d*
Primary outcomes					
ISI	−6.96 (6.39)	−1.63 (3.50)	3.73	<.0001	−1.08
ASIQ	−6.78 (7.21)	−3.59 (8.76)	1.39	.17	−0.40
Suicide risk correlates					
BDI-II	−8.48 (8.66)	−4.33 (6.78)	1.9	0.06	−0.55
PCL-5	−10.26 (14.89)	−5.04 (9.96)	1.48	.15	−0.43
BAI	−4.57 (7.30)	−2.44 (8.25)	0.95	.34	−0.28
CDE Hopelessness^a^	−0.48 (1.34)	0.33 (0.92)	428	.02	−0.55
CDE Hostility	−2.17 (4.56)	−0.22 (4.77)	1.47	.15	−0.43
CDE Belongingness^b^	−0.73 (5.03)	−0.22 (5.44)	0.35	.73	−0.10

	SHUTi-improved, *n* (%)	IEW -improved, *n* (%)	Odds ratio (95% CI)	*P*	
BDI-II 17: Irritability	15 (65)	11 (41)	2.67 (0.75, 10.14)	.10	
BDI-II 19: Concentration	11 (48)	6 (22)	3.13 (0.81, 13.24)	.08	
BDI-II 11: Agitation	12 (52)	14 (52)	1.01 (0.29, 3.55)	1	

ASIQ, Adult Suicidal Ideation Questionnaire; BDI-II, Beck Depression Inventory, second edition; BAI, Beck Anxiety Inventory; CDE, Military Suicide Research Consortium Common Data Elements; IEW, Insomnia Education Website; ISI, Insomnia Severity Index; PCL-5, PTSD Checklist for DSM-5; SHUTi = Sleep Healthy Using the Internet. ^a^Wilcoxon rank sum test and robust Cohen's d were used for hopelessness scale given small range in values ^b^Higher score indicates better health, baseline - post intervention score measuring improvement in symptom

### Effect of SHUTi on suicide risk correlates

A significant between-group difference between SHUTi vs. IEW was observed for the MSRC CDE Hopelessness score (*W* = 428, *P* = .02, Fig. 1C and Table 2) with a medium effect size (robust *d* = −0.55). Medium, but non-significant effect sizes for SHUTi vs. IEW were observed for the BDI-II total (*t*_48_ = 1.9, *P* = .06, *d* = −0.55, Fig. 1D and Table 2), BDI-II 17 Irritability (OR = 2.67, 95% CI: 0.75–10.14, *P* = .10, Table 2) and BDI-II 19 concentration (OR = 3.13, 95% CI: 0.81–13.24, *P* = .08, Table 2). Small-medium sized effects, but not significant group differences were observed for the improvement of PCL-5 (*t*_48_ = 1.48, *P* = .15, *d* = −0.43, Fig. 1E and Table 2) and MSRC CDE Hostility (*t*_48_ = 1.47, *P* = .15, *d* = −0.43, Fig. 1F and Table 2). A small but non-significant effect size of BAI was detected between SHUTi and IEW (*t*_48_ = 0.95, *P* = .34, *d* = −0.28, Table 2). There were non-significant group differences for the improvement of MSRC CDE Belonging and BDI-II 11 Agitation (Table 2). Findings remained consistent when adjusting for baseline scores using linear models. In general, the SHUTi group showed greater within-group improvement across suicide risk correlates than the IEW group (Supplementary Table S1).

## Discussion

Despite the empirical association between insomnia and suicide risk and arguments for the role of insomnia interventions in suicide prevention approaches, to our knowledge, no study has examined the impact of internet- or mobile-based CBT-I intervention in mitigating insomnia severity, SI, and other suicide risk correlates in a high-risk veteran sample. Consistent with our hypothesis, in this secondary data analysis, participants with elevated baseline SI who were randomized to receive SHUTi experienced greater improvement in insomnia symptom severity compared to participants randomized to the IEW. The observed robust effect is consistent with the large effect size observed in face-to-face brief CBT for veterans with insomnia and SI [45] and extends that finding by demonstrating that veterans with elevated SI can benefit from an unguided iCBT-I. It is our hope that this secondary data analysis, along with work by others [45, 66], can be used to support ongoing insomnia intervention work in high-risk cohorts, including veterans.

In partial support of our hypothesis, there was a non-significant, but small-medium effect size between groups on SI, a finding similar to that observed in the proof-of-concept RCT of brief CBT-I in veterans seeking primary care services [45]. In the parent RCT of the current study, there were no inclusion criteria regarding past or current SI [67]. As such, in this secondary analysis of a high-risk cohort, there was a limited range of ASIQ scores, making it challenging to detect improvement in SI given that most participants reported “moderate” levels of SI. In a larger sample, methodologically designed for veterans with elevated and varying levels of SI, we might anticipate larger effect sizes. Given that there was a within-group significant small effect size change on the ASIQ for the IEW group, it may be possible that involvement in the RCT, including having access to psychoeducation on sleep, may have had a positive effect on participants’ mood and SI. Although this may not be a clinically or functionally meaningful change in SI for the IEW group, this may have made it even more challenging to detect an effect between groups. Given the dynamic nature of the association between insomnia and SI, there are likely multiple pathways to improvement within a high-risk cohort. For example, after successfully improving insomnia symptom severity, veterans with briefer histories of SI (e.g. recent SI only in the context of insomnia disorder) vs. veterans with longer histories of SI (e.g. chronic SI) may require differing amounts of time to experience a reduction in SI. To best inform suicide prevention approaches, future research designed to a priori examine our research question is warranted, as are approaches that enable researchers to understand differing pre- and post-intervention recovery trajectories.

We included suicide risk correlates in this secondary data analysis to yield preliminary data to guide the development of future iCBT-I trials examining indicated interventions for at-risk veteran populations. Except for hopelessness, the suicide risk correlates between-group analyses were not significant, contrary to our hypothesis. The effect sizes from some of these analyses, however, suggest candidate variables worth highlighting. First, given the association between insomnia and depression, the medium between-group effect size for depression is consistent with past literature [86]. Further, it is not surprising that three symptoms of depression, hopelessness, irritability, and concentration [87], would also improve. Improvements in concentration may also be associated with cognitive functioning improvement following iCBT-I [88]. For example, reduced total sleep time, a clinical feature of insomnia, has been associated with impaired decision-making [89] in individuals with SI and poor sleep (e.g. short sleep duration) has been associated with lower scores on objective measures of cognitive functioning [90]. In addition to objective cognitive changes, improvements in insomnia may also improve subjective memory functioning [90]. Finally, consistent with previous insomnia treatment studies (reviewed in [28, 91]) small-medium, non-significant effect sizes were also observed for PTSD symptoms, suggesting PTSD symptoms may be a suicide risk correlate that mechanistically improves following iCBT-I, whereas anxiety, belongingness, agitation, and hostility may be suicide risk correlates less proximal to the insomnia-suicide pathway or that require additional intervention or time to improve.

Although this is the first known examination of insomnia symptoms and suicide risk correlates in a high-risk veteran sample who received iCBT-I, several limitations are important to highlight. As previously mentioned, this is a secondary data analysis that was not designed for this specific examination. Most of our included correlates were derived from psychometrically validated assessments (i.e. insomnia severity, SI, depressive symptoms, PTSD symptoms, anxiety symptoms, MSRC CDEs), but others relied on single-item assessments (e.g. agitation, irritability, concentration) that do not capture all objective and subjective facets of these constructs. Because of the preliminary nature of these analyses, we focused on effect sizes; future studies are necessary to replicate these effect sizes in representative samples with ample power. Most participants in the defined cohort identified as male, White, and non-Hispanic. The lack of representativeness of other identity variables in this sample is a significant limitation; and thus, warrants replication in RCTs with diverse participants that include underrepresented populations.

## Conclusion

In a veteran cohort reporting clinically significant insomnia severity and SI, engagement in an iCBT-I was associated with improvement in insomnia symptom severity and hopelessness, compared to an IEW control. Non-significant, but promising between-group effect sizes were observed across insomnia and some suicide correlates (SI, depressive symptoms, concentration, PTSD symptoms, hostility, irritability), suggesting that these important clinical outcomes can be improved with access to and engagement in an iCBT-I. Improving our understanding of the mechanisms underlying the association between insomnia and suicide risk, in models that include self-administered evidence-based treatment approaches and important suicide correlates, especially in high-risk samples, are necessary to continue to advance and implement novel suicide prevention approaches.

## Supplementary data

Supplementary material is available at *Translational Behavioral Medicine* online.

ibae032_suppl_Supplementary

## Data Availability

Data collected for this study, including de-identified participant data and a data dictionary, will be made available to those with approval and/or signed data access agreements for scientific investigations. Data sharing must comply with sponsoring agency regulations. Requests may be sent via email to the corresponding author.

## Welfare of Animals

This article does not contain any studies with animals performed by any of the authors.

## Transparency Statements

*Study Registration*: The parent study of this secondary data analysis was pre-registered at ClinicalTrials.gov NCT03366870; https://clinicaltrials.gov/ct2/show/NCT03366870. This study’s secondary data analysis was not formally registered. *Analytic Plan Pre-Registration*: The analysis plan for this secondary data analysis was not formally pre-registered. *Analytic Code Availability*: Analytic code used to conduct the analyses presented in this study is not available in a public archive. They may be available by emailing the corresponding author. *Materials Availability*: Materials used to conduct the study (e.g. study protocol, informed consent form) may be requested by sending an email to the corresponding author.
