# Supplementary material for: Impact of an internet-based insomnia intervention on suicidal ideation and associated correlates in veterans at elevated suicide risk
Source: Transl Behav Med. 2024 Jun 12;14(11):673–83. doi: 10.1093/tbm/ibae032 (PMC11568844; doi:10.1093/tbm/ibae032)
Supplement: ibae032_suppl_Supplementary [file ibae032_suppl_supplementary.docx]

|  | **SHUTi (n=23)** | | | **IEW (n=27)** | | |
| --- | --- | --- | --- | --- | --- | --- |
|  | **test statistic** | **p** | **d** | **test statistic** | **p** | **d** |
| **ISI** | -5.22 | <.0001 | -1.09 | -2.42 | 0.01 | -0.47 |
| **ASIQ** | -4.51 | <.0001 | -0.94 | -2.13 | 0.02 | -0.41 |
| **BDI-II** | -4.70 | <.0001 | -0.98 | -3.32 | 0.001 | -0.64 |
| **PCL-5** | -3.31 | 0.002 | -0.69 | -2.63 | 0.01 | -0.51 |
| **BAI** | -3.00 | 0.003 | -0.63 | -1.54 | 0.07 | -0.30 |
| **CDE Hopelessness^1^** | 36.5 | 0.06 | -0.39 | 100 | 0.96 | 0.36 |
| **CDE Hostility** | -2.29 | 0.02 | -0.48 | -0.24 | 0.41 | -0.05 |
| **CDE Belongingness^2^** | -0.70 | 0.24 | -0.15 | -0.21 | 0.42 | -0.04 |

**Table S1.**Within group changes in clinical outcomes post intervention, reporting test statistics from the within group improvement analyses using one-sided t test or one-sided Wilcoxon signed-rank test on the improvement of scores. Note, SHUTi = Sleep Healthy Using the Internet; IEW = Insomnia Education Website; ISI = Insomnia Severity Index; ASIQ = Adult Suicidal Ideation Questionnaire, PCL-5 = PTSD Checklist for DSM-5; BDI-II = Beck Depression Inventory, second edition; BAI = Beck Anxiety Inventory; CDE = Military Suicide Research Consortium Common Data Elements.
